# Tumor cell-derived hyaluronan fragments induce endocytosis of S1PR1 to promote lymphangiogenesis through LYVE-1-Src pathway

**DOI:** 10.7150/jca.104309

**Published:** 2025-01-27

**Authors:** Mengying Jiang, Dandan Chen, Zhangrun Xu, Yiwen Liu, Cuixia Yang, Guoliang Zhang, Qian Guo, Feng Gao, Yiqing He, Yan Du

**Affiliations:** 1 Department of Molecular Biology, Shanghai Sixth People's Hospital Affiliated to Shanghai Jiao Tong University School of Medicine, 600 Yishan Road, Shanghai 200233, China.; 2 Department of Clinical Laboratory, Shanghai Sixth People's Hospital Affiliated to Shanghai Jiao Tong University School of Medicine, 600 Yishan Road, Shanghai 200233, China.

**Keywords:** Hyaluronan fragments, lymphangiogenesis, S1PR1, endocytosis, LYVE-1, tumor

## Abstract

Sphingosine-1-phosphate receptor-1 (S1PR1), a G protein-coupled receptor, has been reported to be involved in lymphangiogenesis. Degradations of extracellular matrix (ECM) are recognized as dynamic modulators in regulating the formation of new lymphatic vessels. However, little research has studied the ECM on S1PR1 in the regulation of lymphatic endothelial cells (LECs) in tumor lymphangiogenesis. Here we attempt to investigate hyaluronan fragments abundant in tumor microenvironment (TME) on S1PR1 in new lymphatic vessel formation. First, we verified that low molecular weight hyaluronan (LMW-HA) derived from tumor cells could promote LECs migration and capillary-like tube formation. Then, we demonstrated that S1PR1 on LECs underwent internalization into the endoplasmic reticulum in response to LMW-HA treatments. Notably, the S1PR1 endocytosis could upregulate lymphangiogenesis. Next, we found that the ablation of lymphatic vessel endothelial hyaluronan receptor 1 (LYVE-1) could attenuate the S1PR1 endocytosis, implying a novel role of LMW-HA/LYVE-1 in the S1PR1 cycling pathway. Furthermore, we identified that LMW-HA/LYVE-1 interaction could activate Src kinase which in turn upregulates S1PR1 tyrosine phosphorylation, resulting in S1PR1 endocytosis. Collectively, our findings suggested that hyaluronan fragments in TME could induce S1PR1 internalization in LECs, leading to lymphangiogenesis promotion.

## Introduction

Lymphangiogenesis, a process that generates new lymphatic vessels from pre-existing conduits, is of great significance for tumor metastasis^1^. G protein-coupled receptor S1PR1, one receptor of Sphingosine-1-phosphate (S1P), is a novel regulator of lymphangiogenesis with conflicting biological activities. Several studies showed that S1PR1 could promote lymphangiogenesis under the stimulation of sphingosine-1-phosphate (S1P) through activating the Gi-phospholipase C-Ca^2+^ signaling pathway or NF-kB pathway^2^, ^3^. In contrast, others reported that S1PR1 receptor itself could inhibit lymphangiogenesis by strengthening the formation of tight connections between lymphatic endothelial cells (LECs)^4^. When the S1PR1 receptor was genetic inhibited in mice, lymphatic vessel budding capacity was enhanced, and lymphatic networks could be expanded^3^. These studies suggest that the S1PR1 pathway in lymphangiogenesis may be part of a complicated regulatory network. In particular, the regulatory mechanisms of S1PR1 in tumor lymphangiogenesis remain elusive.

Newborn lymphatic endothelial cells (LECs) are in direct contact with the interstitial matrix and lack a basement membrane^5^. Extracellular matrix (ECM) in the tumor microenvironment (TME) frequently undergoes dynamic remodeling along with tumor progression^6^. Consequently, the aberrantly altered matrix in the peri-tumoral region may exert significant effects on tumor lymphangiogenesis by directly influencing LECs^5^. Previous investigations have established that hyaluronic acid (HA, ~10^7^ Da), a predominant constituent of the tumor extracellular matrix (ECM), undergoes degradation by hyaluronidases and other factors like reactive oxygen and nitrogen, yielding low molecular weight HA (LMW-HA) fragments^7^. We and other studies have shown that these fragments are involved in tumor lymphangiogenesis by binding to lymphatic vessel endothelial hyaluronan receptor 1 (LYVE-1), which is an identified 41% homolog of the cluster of differentiation 44 (CD44) glycoprotein and is largely restricted to the endothelial cells as a lymphatic-specific receptor for HA^7^. However, the subsequent downstream mechanisms remain inadequately characterized.

Recently, S1PR1 has been shown to associate with CD44^8^. Sphingosine-1-phosphate receptor 3 (S1PR3), another S1P receptor, has also been reported to bind to CD44 and LYVE-1 in vascular/lymphatic endothelial cells, respectively^7^, ^8^. Based on these, it is plausible to hypothesize a novel interaction between S1PR1 and LYVE-1 and S1PR1 may implicate in LYVE-1-mediated lymphangiogenesis, a process critical to the development and maintenance of the lymphatic system. Previous studies indicated that S1PR1 localization on the cell membrane is required to maintain vascular homeostasis and that S1PR1 internalization exerts an important role in destroying endothelial barrier function^9^. S1PR1 endocytosis mediated by S1PR1 phosphorylation has been shown to be a common mechanism of inflammatory mediators such as TNF-α^9^. ECM components and their degradative products such as LMW-HA are another kind of active stimulator in TME^10^. However, few studies have focused on whether TME-dynamically remodeled ECM components could regulate S1PR1 intracellular distribution, or its role in influencing the formation of new lymphatic vessels in tumors.

This study was aimed to investigate the factors affecting the localization of S1PR1 on the surface of lymphatic endothelial cells and its role in tumor lymphangiogenesis. We first observed that S1PR1 could translocate from LEC cell surface to endoplasmic reticulum under the stimulation of LMW-HA, thus triggering tumor lymphangiogenesis. Then, we found that S1PR1 could form complexes with LYVE-1 and LYVE-1 knockout could reduce S1PR1 internalization. Furthermore, we showed that Src signaling was required for LMW-HA-LYVE-1-induced S1PR1 endocytosis. This study has unveiled a novel finding that tumor cell-derived LMW-HA-induced S1PR1 endocytosis could trigger lymphangiogenesis, in which LYVE-1-mediated enhancement of Src phosphorylation may be responsible for the underlying mechanism.

## Materials and Methods

### Cell culture and reagents

Human lymphatic endothelial cells (HLECs) were obtained from the PromoCell (C-12216, Heidelberg, Germany). Mouse lymphatic endothelial cells (SVEC4-10) were purchased from the American Type Culture Collection. Mouse breast carcinoma cells (4T1) were purchased from the Cell Bank of the Type Culture Collection of the Chinese Academy of Sciences (Shanghai, China). HLECs were cultured in EGM-2 (CC-3202, Lonza, BSL, Switzerland). And HLECs with passage numbers ranging from 4 to 7 were used for the experiments. SVEC4-10 and 4T1 cells were cultured in Dulbecco's modified Eagle's medium (BasalMedia, Shanghai, China) and RPMI 1640 medium (BasalMedia, Shanghai, China) respectively, supplemented with 10% fetal bovine serum (FBS) (BasalMedia, Shanghai, China), 100 U/mL penicillin and 100 mg/mL streptomycin. 4T1^HAS2-/-^ cells were prepared as previously described^6^. All cells were tested mycoplasma-negative and cultured in a humidified atmosphere under 5% CO2. Agonist Sphingosine-1-phosphate (S1P, HY-108496, MedChemExpress, NJ, USA) and inhibitors dynasore (HY-15304, MedChemExpress, NJ, USA), PP2 (T6266, TOPSCIENCE, Shanghai, China) were used.

### *In vivo* experiments

All animal experiments were approved by the Animal Care and Use Committee of Shanghai Jiao Tong University Affiliated Sixth People's Hospital. Female 7-week-old-Balb/c mice were obtained from Shanghai SLAC Laboratory Animal Co., Ltd. 5 × 10^4^ 4T1 cells or 4T1^HAS2-/-^ cells in 100 μL of PBS were separately injected into the left fourth mammary fat pad of random BALB/c female mice (n=5, respectively), and the control group (n=5) was injected with equal volume of phosphate buffered saline (PBS) only. 14 days after cancer cells inoculation, when the primary tumor diameter reached 1 cm, mice were sacrificed. The tumor tissues in the 4T1 and 4T1^HAS2-/-^ groups and the mammary fat pad in the control group were collected for lymphangiogenesis analysis.

### Immunohistochemistry (IHC)

Immunohistochemistry of mouse tissue slices was performed on formalin-fixed, paraffin-embedded sections as previously described^6^. Anti-LYVE1 (ab14917, Abcam, Cambridge, MA, USA) was used at a dilution of 1:500. After incubation with horseradish peroxidase-conjugated secondary antibody for 20 min at 37°C, the tissue sections were treated with 3-Amino-9-Ethylcarbazole and stained with hematoxylin. The staining was visualized using a light microscope.

### Conditioned media (CM)

For conditioned media, 4T1 or 4T1^HAS2-/-^ cells (3 × 10^6^) were cultured to 50% ~ 60% confluency in T75 tissue culture flasks with normal growth media, then washed in PBS twice, and incubated in 16 mL serum-free media. After 48 h incubation, 4T1 or 4T1^HAS2-/-^ cells were lysed for protein concentration detection. The collected supernatant was centrifuged and filtered through 0.22 μm syringe filters (Corning, San Diego, CA, USA). The most of resulting tumor-conditioned media were stored in aliquots at -80 °C for future use and the other was used for HA concentration determination by chemiluminescence immunoassay (New Industries Biomedical Engineering, Shenzhen, China). The relative amount of HA was calculated as follows: HA concentration/protein concentration of 4T1 or 4T1^HAS2-/-^ cells.

### Immunofluorescent staining

SVEC4-10 cells seeded on 8-well glass-bottomed dishes (155382, Thermo Fisher, Waltham, MA, USA) were fixed with 4% paraformaldehyde for 10 min and permeabilized with 0.1% Triton X-100 in PBS for 10 min, then blocked in 3% bovine serum albumin (BSA) for 1 h. The cells were then incubated with primary antibodies at 4℃ overnight in 1×PBS supplemented with 3% BSA and then with secondary antibodies for 1 h at room temperature. Images were obtained and analyzed under confocal microscopy (Nikon A1, Tokyo, Japan). The primary anti-BiP antibody (sc-13539) was purchased from Santa Cruz Biotechnology (Dallas, TX, USA) and the anti-S1PR1 antibody (55133-1-AP) was obtained from Proteintech (Wuhan, HB, China). The primary anti-LYVE-1 antibody (MAB2125) was obtained from R&D Systems (Minneapolis, MN, USA). Alexa Fluor 594- and 488-conjugated IgG were used as the secondary antibodies (ab150112, ab150081, Abcam, Cambridge, MA, USA). Phalloidin (40762ES75) was purchased from Yeasen (Shanghai, China) which indicates cell morphology.

### siRNA transfection

To knock down S1PR1 or LYVE-1, SVEC4-10 cells at ~ 80% confluency were transfected with 100 nM S1PR1/LYVE-1 siRNA or negative control siRNA (RIBOBIO, Guangzhou, GD, China**)** by using Lipofectamine RNAiMax Transfection Reagent (13778030, Invitrogen, Carlsbad, CA, USA). All experiments were performed according to the manufacturer's protocol. 6-8h after transfection, the medium was discarded and replaced with normal growth media with or without LMW-HA. All validation and functional experiments were conducted effectively within 72h. The siRNA sequences were listed as follows: LYVE-1 siRNA (5′-CAACGCTAATGAAGAATCA-3′), S1PR1 siRNA (5′-GCCGCAGCAAATCAGACAA-3′), and scramble negative control siRNA (5′-UUCUCCGAACGUGUCACGU-3′).

### LYVE-1 CRISPR/Cas9 KO stable clone

The mouse LYVE-1 CRISPR/Cas9 KO plasmid and control CRISPR/Cas9 plasmid were purchased from GENECHEM (Shanghai, China). Knocking out LYVE-1 expression in SVEC4-10 was performed according to the manufacturer's instructions. SVEC4-10 cells (6 × 10^4^) were plated on 24-well plates. After 24 h incubation, the cells were transfected with CRISPR/Cas9 plasmids according to the manufacturer's protocol when reaching 80% confluence. The ratio of the target plasmid or negative control one to the transfection reagent was 0.5 μg/1 μL. After 15 min of incubation at room temperature, the complexes were added to the cells carefully. 24h after transfection, the medium was replaced with normal medium. Puromycin (2 μg/mL, Sigma Aldrich, St. Louis, MO) was used subsequently to screen stably transduced cells over 15 days. Finally, the knock-out efficiency was verified by western blotting assay.

### Quantitative real-time PCR

Total RNA was extracted from cultured cells using RNAiso Plus (Takara, Kusatsu, Shiga, Japan). The RNA of all samples was reverse-transcribed to complementary DNA with genomic DNA eraser (Takara, Kusatsu, Shiga, Japan). Then complementary DNA was amplified with a SYBR Green PCR Kit (Takara, Kusatsu, Shiga, Japan) on an ABI 7500 system sequence detection system, with the primers (Supplementary Table 1). β-actin (ACTB) was amplified as an internal control.

### Tube formation assay

Matrigel (3445-010-01, R&D Systems, Minneapolis, MN, USA), thawed on ice at 4°C overnight, was loaded into each well of a pre-cooled 96-well plate, and the plate was incubated at 37 °C for 30 min. SVEC4-10 and HLECs were resuspended in the medium, then seeded at 4 × 10^4^ cells/well onto the Matrigel. After 6 h incubation, the viable cells were visualized with an inverted microscope (Olympus) with a digital camera (Cannon). The number of junctions was measured by using Image J software (version 1.53p).

### Wound healing assay

2 × 10^4^ SVEC4-10 cells were seeded into a 24-well plate and transfected with siRNA on the third day. After 36 h, cells were detached with trypsin, counted, and centrifuged for 5 min at 1000 rpm. The cell pellet was dissolved in a medium to generate a concentration of 1.5 × 10^5^ cells/mL. 100 μL of cell suspension was applied into each well of the ibidi Culture-Insert 2 well system (81176, Ibidi, Gräfelfing, Germany) positioned in a 6-well plate. The culture insert was removed, and the cells were rinsed with medium and treated with 10 μg/mL LMW-HA or fresh growth medium. Photographs of wounds were taken at regular intervals over 12 hours. For every time point, three pictures were taken and evaluated. The wound closure of the monolayer was imaged at the indicated time by an inverted microscope (Olympus) with a digital camera (Cannon). The wound area was measured by using Image J software (version 1.53p). The wound closure rates were calculated as follows: (the initial wound area - the wound area at an indicated time) divided by the initial wound area.

### Western blotting

Western blotting analysis was performed as previously described^11^. The primary antibodies used in the study were as follows: anti-S1PR1 (55133-1-AP, Proteintech, Wuhan, HB, China), anti-LYVE-1 (51011-1-AP, Proteintech, Wuhan, HB, China), anti-phospho-Src (Tyr416) (6943S, Cell Signaling Technology, Boston MA, USA), anti-phospho-Tyr (PY99) (sc-7020, Santa Cruz Biotechnology, Dallas, TX, USA). We used β-actin (P30002, Abmart, Shanghai, China) as internal controls. The PVDF membranes were incubated with anti-mouse (M21001S, Abmart, Shanghai, China) or anti-rabbit (M21002S, Abmart, Shanghai, China) secondary antibodies for 1 h. Immunoblotting was visualized using an enhanced chemiluminescence solution (Pierce, Pierce, MO, USA), and the ImageQuant LAS 4000 mini (General Electric Company, Boston, MA, USA) was used to detect protein expression.

### Immunoprecipitation

For immunoprecipitation analysis, SVEC4-10 cells were washed with ice-cold PBS and immediately lysed in a modified radioimmunoprecipitation assay buffer (KGP704, KeyGEN BioTECH, Nanjing, JS, China). Lysates were incubated with Normal IgG (1:100) (Beyotime, Shanghai, China) or anti-S1PR1 (1:100) antibodies overnight followed by the addition of Protein A/G Beads (York Biotech, Shanghai, China) to pull down the immunocomplexes^9^. Proteins were separated by SDS-PAGE and immunoblotted using indicated antibodies. To avoid interference from IgG, membranes were then incubated with an HRP-conjugated Veriblot for IP Detection Reagent (ab131366, Abcam, Cambridge, MA, USA) for 1 h, following which the bands were subsequently visualized using the enhanced plus chemiluminescence assay (SQ201, Epizyme, Shanghai, China). Measurement of the bands was conducted on an ImageQuant LAS 4000 mini.

### Statistical analysis

All statistical analyses were carried out using GraphPad Prism Software (GraphPad Software Inc., San Diego, CA, USA). The statistical significance of differences between two different groups and multiple groups was determined by Student's t-test and one-way ANOVA respectively. A *p*-value <0.05 was considered statistically significant. All assays were performed at least three independent experiments.

## Results

### Tumor cell-derived LMW-HA could promote lymphangiogenesis

**Figure 1:**
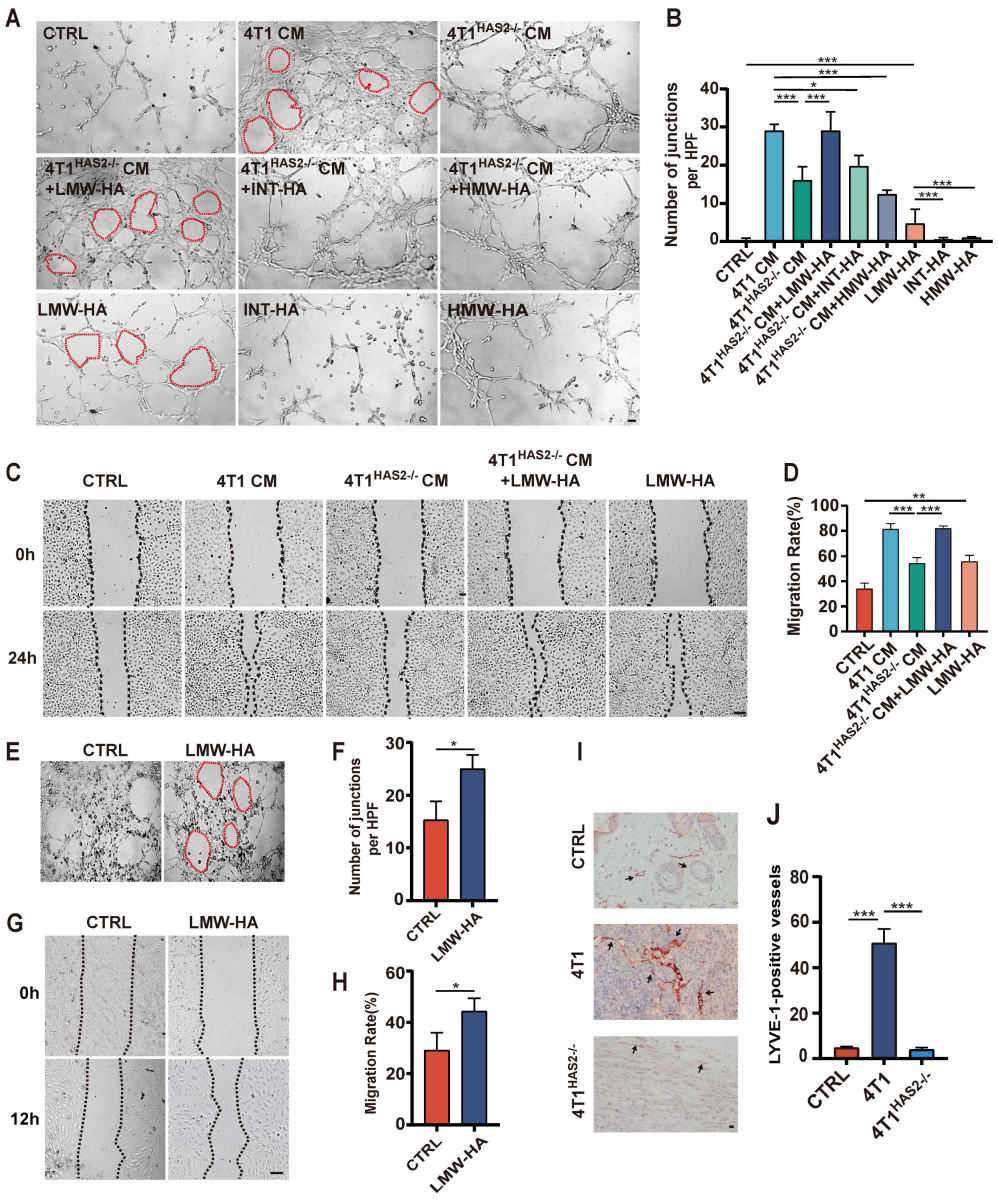
**Tumor cell-derived hyaluronan fragments promote lymphangiogenesis.** (A, B) Representative images (A) and quantitative analysis (B) of the tube formation abilities of SVEC4-10 cells incubated with 4T1 cells CM, LMW-HA (10 μg/mL) / INT-HA (5 μg/mL) / HMW-HA (10 μg/mL) with or without 4T1^HAS2-/-^cells CM and the corresponding control cells. The scale bar represents 50 μm. (C, D) Representative images (C) and quantitative analysis (D) of the wound healing assays of SVEC4-10 cells stimulated by 4T1 cells CM, 4T1^HAS2-/-^cells CM, 4T1^HAS2-/-^cells CM+LMW-HA, LMW-HA and the corresponding control cells for 24h. The scale bar represents 50 μm. (E-G) Representative images (E, G) and quantitative analysis (F, H) of the tube formation and wound healing assays of HLECs treated with LMW-HA (10 μg/mL) and the control cells for 6h and 12h, respectively. (I, J) Representative staining images (I) and quantitative analysis (J) of breast sections for LYVE-1 (red) positive lymphatic vessels in the 4T1 (n=5), 4T1^HAS2-/-^ (n=5) and the control group (n=5). The scale bar represents 50 μm. The data are presented as the mean ± SEM of three independent experiments. *p < 0.05, **p < 0.01, ***p < 0.001 in One-way ANOVA or students' t test.

Tumor cells are regarded as one major source of LMW-HA in tumor microenvironment (TME)^10^. Therefore, we used 4T1 conditioned media (CM) in which LMW-HA was abundant to study the effect of tumor cells-derived LMW-HA on lymphangiogenesis^6^. Hyaluronan (HA) is dynamically remodeled in TME and is reported to be closely related to tumor lymphatic metastasis by inducing lymphangiogenesis^11^. Our results showed that the in vitro capillary-like tube formation and the migration capacity of lymphatic endothelial cells were significantly increased after co-culturing with the CM of 4T1 cells (Fig. 1A-D). Since hyaluronan Synthase 2 (HAS2) is the main hyaluronic acid synthetase in the 4T1 cells^12^, we knocked out HAS2 in 4T1 cells to reduce the amount of LMW-HA. Our data indicated that the ability of LECs to migrate and form capillary tubes was decreased by reducing tumor cell-derived LMW-HA (Fig. S1, 1A-D). Next, we also found that adding LMW-HA to the supernatant of 4T1^HAS2-/-^ CM could restore its ability to promote tube formation, while intermediate-sized hyaluronan (INT-HA) and high molecular weight hyaluronan (HMW-HA) did not (Fig. 1A, B). Further, we observed that the addition of LMW-HA alone can also stimulate lymphatic vessel growth (Fig. 1A-H). In addition, we have demonstrated that 4T1 cells are more capable of inducing tumor lymphangiogenesis *in vivo* than 4T1^HAS2-/-^ cells (Fig. 1I, J). Taken together, our data suggested that tumor cell-derived LMW-HA could stimulate new lymphatic vessel formation.

### S1PR1 endocytosis occurred in LEC cells after LMW-HA stimulation

**Figure 2:**
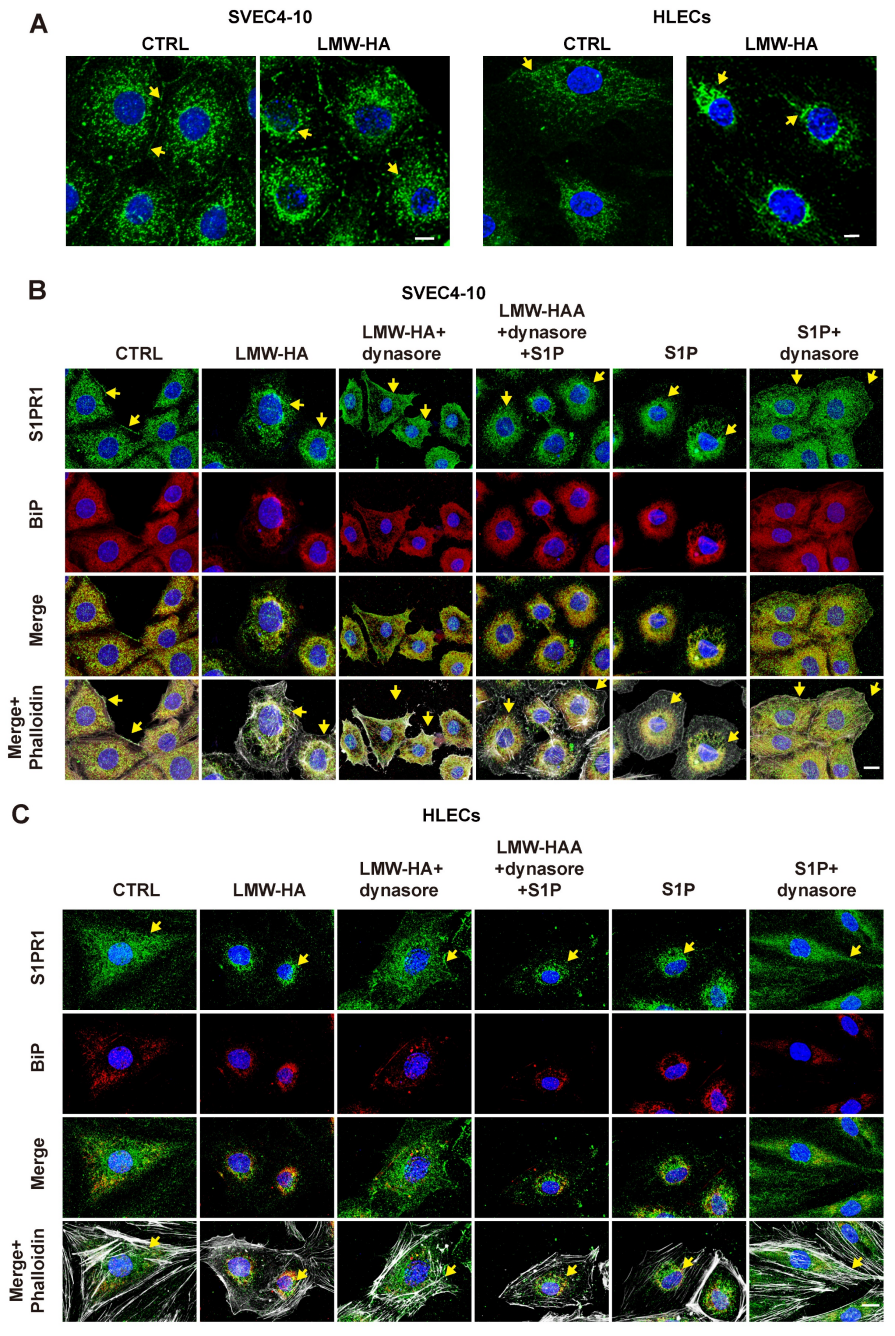
** S1PR1 endocytosis occurred in LEC cells after LMW-HA stimulation.** (A) Confocal images of S1PR1 (green) in SVEC4-10 and HLECs treated in the presence or absence of LMW-HA (10 μg/mL) treatment for 2 h. (B, C) Confocal images of S1PR1 (green), BiP (red, an endoplasmic reticulum marker) and phalloidin (white, indicating cell morphology) in SVEC4-10 cells (B) and HLECs (C). Cells were treated with the following conditions: LMW-HA (10 μg/mL) for 2 hours. LMW-HA + dynasore: cells were pre-treated with dynasore (80 μM) for 3 hours before LMW-HA stimulation for 2 hours. S1P (1μM) for 1 hour as a positive control. S1P + dynasore: Cells were pre-treated with S1P for 1 hour before the addition of dynasore for 3h. Nuclei were stained with DAPI (blue). Representative fluorescent images from three independent experiments were shown. The scale bar represents 10 μm. Arrows (yellow) indicate S1PR1.

Proper subcellular localization of S1PR1 is critical for its regulation of EC function, and the redistribution of S1PR1 is easily regulated by inflammatory factors^9^, which prompted us to further investigate whether the distribution of S1PR1 is also influenced by another active mediator Like LMW-HA and its role in tumor lymphangiogenesis. First, upon LMW-HA stimulation of LECs, we observed a redistribution of S1PR1 from sub-membrane localization to intracellular aggregation, predominantly within the perinuclear region (Fig. 2A). Subsequent immunofluorescence assays employing an endoplasmic reticulum marker binding immunoglobulin protein (BiP) demonstrated that the majority of internalized S1PR1 triggered by LMW-HA, were co-localized with BiP-positive structures within the endoplasmic reticulum (ER) of LECs (Fig. 2B, C). This observation was consistent with the internalization pattern observed with the positive control S1P^9^, thereby confirming the ER as the primary subcellular location for internalized S1PR1 (Fig. 2B, C). In addition, it has been confirmed that the canonical S1PR1 endocytosis is mediated by dynamin and can be strongly inhibited by dynasore (an inhibitor of dynamin)^9^. To elucidate whether the intracellular accumulation of S1PR1 in response to LMW-HA is attributable to dynamin-dependent endocytosis, SVEC4-10 and HLEC cells were exposed to dynasore. Our findings indicated a resurgence of S1PR1 at the plasma membrane after 3 h dynasore treatment, suggesting that LMW-HA-induced S1PR1 internalization is mediated by dynamin (Fig. 2B, C). These results suggest that the distribution of S1PR1 is regulated not only by its ligand S1P, but also by the degradation fragments of HA in the ECM. In this experiment, we also found that similar to the effect of S1P, the ER localization of LMW-HA-stimulated S1PR1 is not due to the accumulation of misfolded proteins, but to the enhancement of classical endocytosis.

### LMW-HA-induced S1PR1 internalization promotes lymphangiogenesis

**Figure 3:**
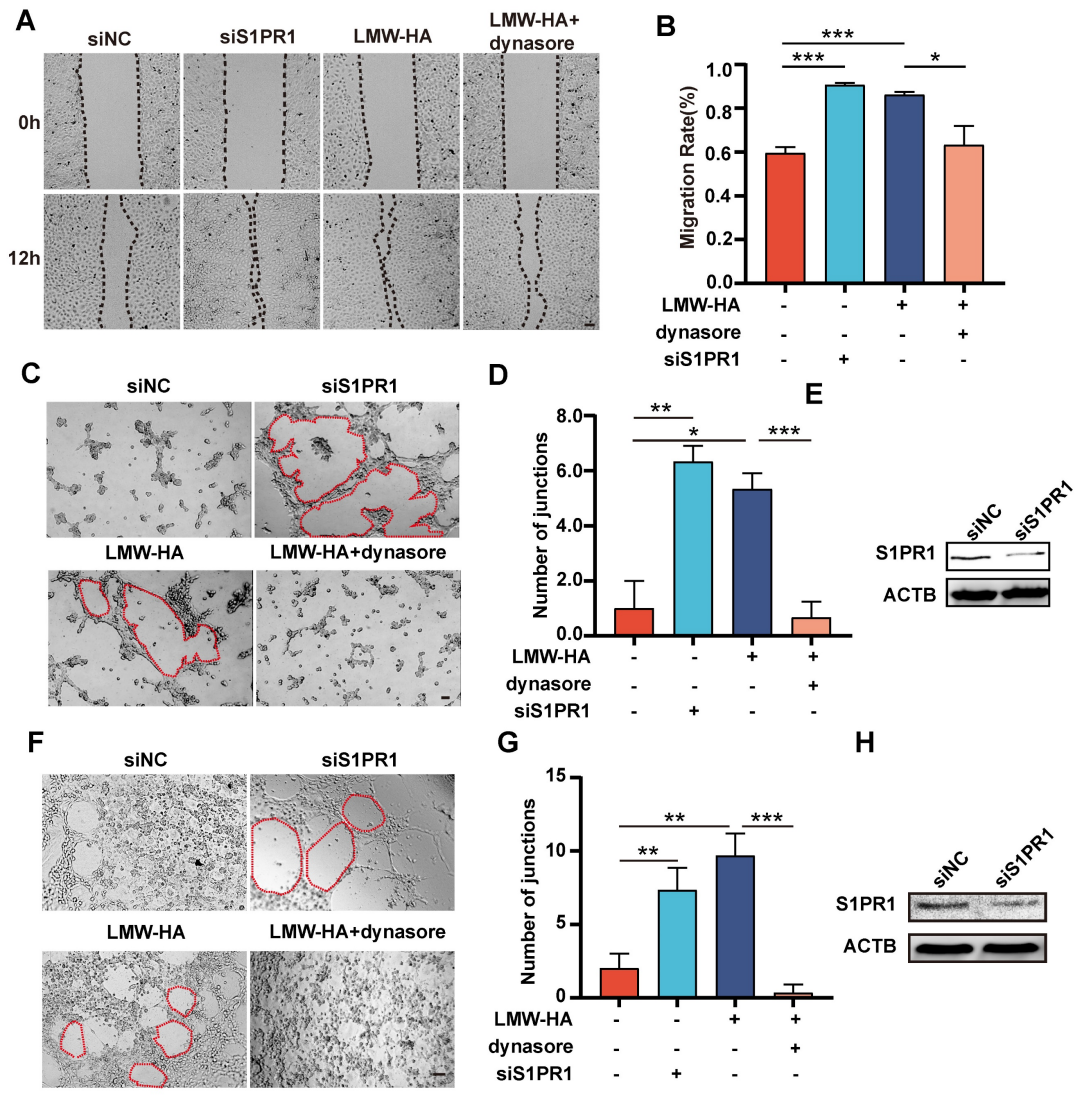
** S1PR1 internalization involves in LMW-HA-induced lymphangiogenesis.** (A-D) Representative images (A, C) and quantitative analysis (B, D) of the wound healing and the tube formation assays of SVEC4-10 cells, respectively. Cells were subjected to LMW-HA treatment, with or without dynasore (an endocytosis inhibitor), and S1PR1 knockdown. The scale bar represents 50 μm. (F, G) Representative images (A) and quantitative analysis (G) of the tube formation assays of HLECs. Cells were treated as same as SVEC4-10 cells (A-D). The scale bar represents 50 μm. (E, H) S1PR1 expression in SVEC4-10 (E) and HLEC cells (H) was detected by western blot analysis after 72 hours of siRNA infection. β-actin (ACTB) was used as the loading control. Data are obtained from at least three independent experiments and represent mean ± SEM. *p < 0.05, **p < 0.01, ***p < 0.001 in One-way ANOVA.

To determine if S1PR1 endocytosis was implicatd in LMW-HA-induced enhancement of lymphangiogenesis, we initiated our investigation by inhibiting S1PR1 internalization with dynasore and then examined its effects on LEC migration and tube formation. Our results indicated that inhibition of S1PR1 endocytosis by dynasore resulted in a significant reduction in LMW-HA-induced LEC migratory and tube formation capacities of SVEC4-10 and HLEC cells (Fig. 3A-G), suggesting that LMW-HA-induced endocytosis of S1PR1 leads to lymphatic network expansion. Furthermore, aligning with previous study^4^, our study also found that down-regulation of S1PR1 by RNA interference could enhance lymphaitc endothelial cell migration and tube formation. Combined with our above results, this study further suggests that membrane-bound S1PR1 has an inhibitory effect on lymphangiogenesis (Fig. 3A-H). Together, these findings propose that LMW-HA induces aberrant S1PR1 cell surface localization, disrupting the inhibitory S1PR1 signaling and consequently promoting lymphangiogenesis.

### S1PR1 directly interacts with LYVE-1 in LECs

**Figure 4:**
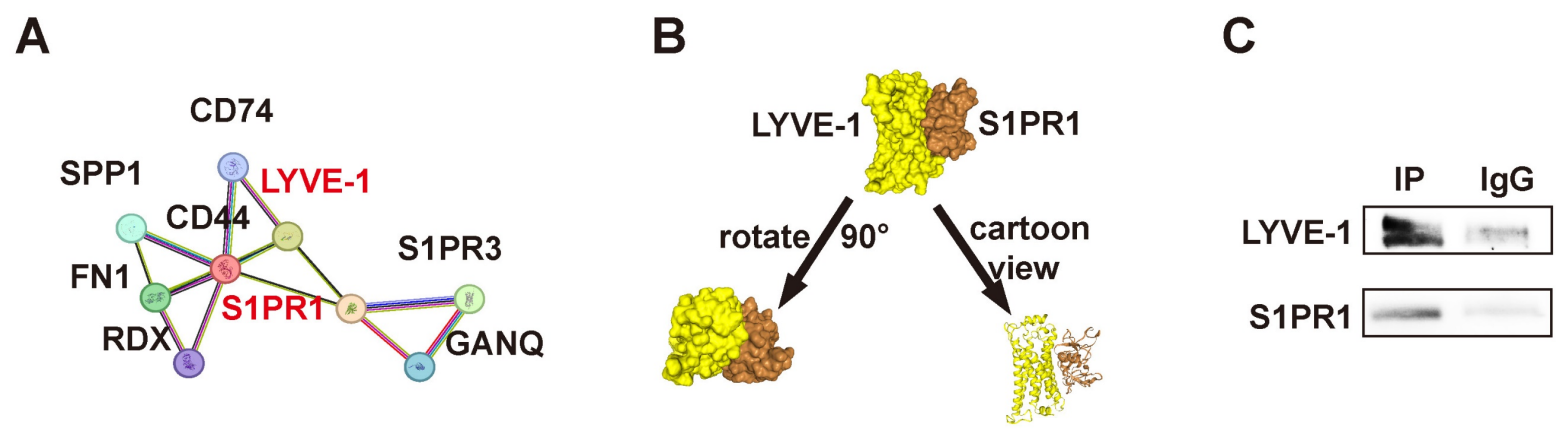
** S1PR1 directly interacts with LYVE-1 in LECs.** (A) The STRING database predicts protein interactions between S1PR1 and LYVE-1. (B) Top-ranked bound conformation of the LYVE-1-S1PR1 complex of S1PR1 and LYVE-1, as predicted by the HDOCK SERVER. Docking Score: -309.97, Confidence Score: 0.9608. (C) Co-immunoprecipitation assay indicates the direct connection of LYVE-1 to S1PR1 in LECs.

Our previous studies have shown that LMW-HA promotes lymphangiogenesis mainly through LYVE-1, but its underlying mechanism has not been fully clarified. Given the established interaction between S1PR1 and CD44, of which LYVE-1 has 41% homolog, and the reported binding of S1PR3 to CD44 and LYVE-1 in vascular and lymphatic endothelial cells, respectively^7^, ^8^, coupled with the observation of colocalization between lymphatic LYVE-1 and S1PR1 in sagittal sections of the mouse thoracic aorta^13^, it is hypothesized that LYVE-1 and S1PR1 may function in a directly or indirectly coordinated manner within lymphatic vessels. In the present study, we first analyzed the protein-protein network of S1PR1-correlated genes from the STRING database (https://string-db.org/) and found that S1PR1 and LYVE-1 were physically and functionally associated (Fig. 4A). Next, the top-ranked bound conformation of the LYVE-1-S1PR1 complex (Docking Score: -309.97, Confidence Score: 0.9608; Fig. 4B) was depicted by the computational protein-protein docking algorithm from HDOCK SERVER (http://hdock.phys.hust.edu.cn/). Subsequently, we used a quantitative polymerase chain reaction assay to determine that both mouse and human LECs express S1PR1(Fig. S2). Then, the binding assay of LYVE-1 to S1PR1 in LECs was conducted by co-immunoprecipitation and the western blot confirmed that LYVE-1 and S1PR1 do have a physical connection (Fig. 4C). These results confirmed that there is close interaction between LYVE-1 and S1PR1 in LECs.

### LYVE-1 is required by LMW-HA-induced S1PR1 internalization

**Figure 5:**
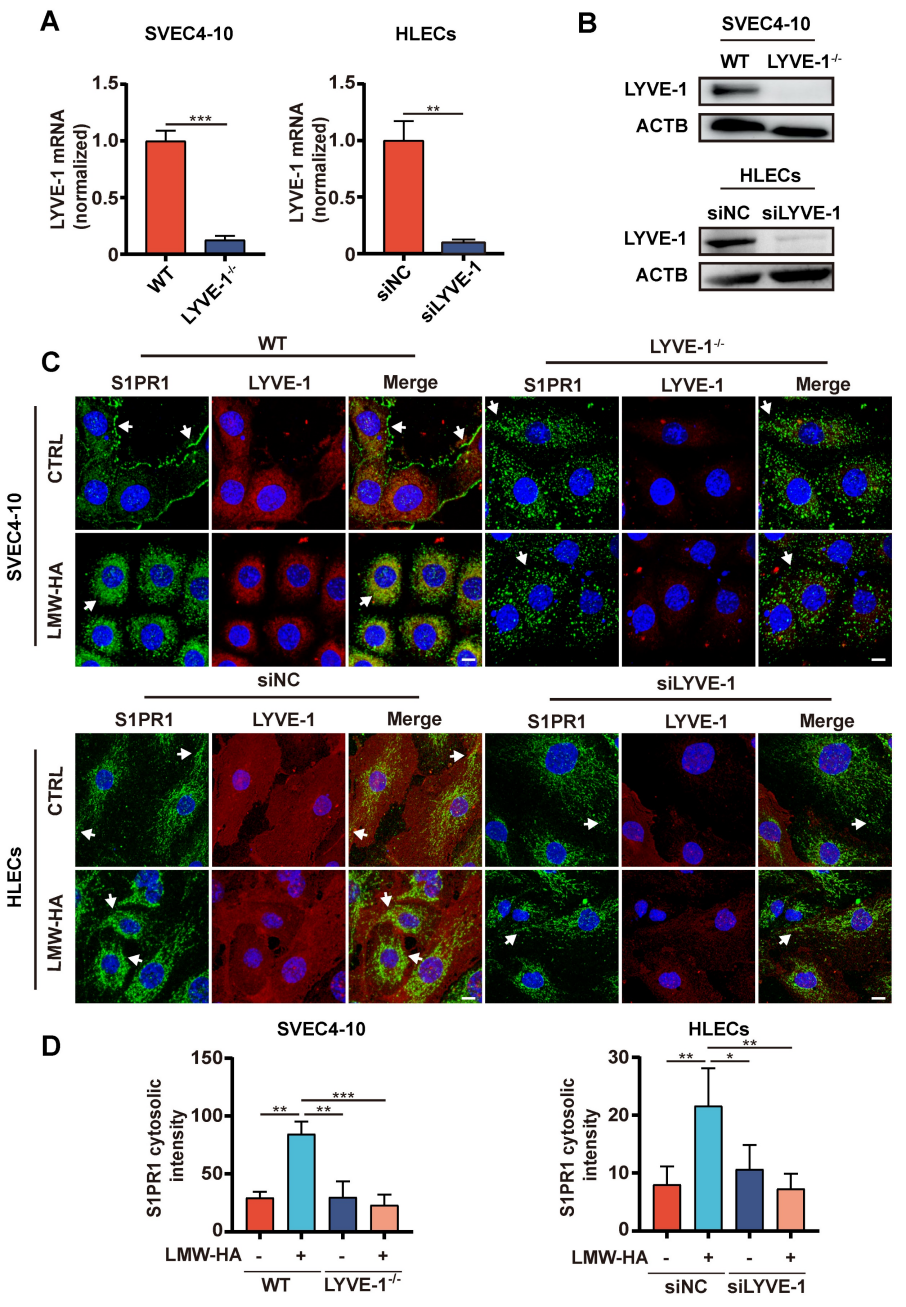
** LYVE-1 is required for the LMW-HA-induced S1PR1 internalization.** (A, B) LYVE-1 mRNA (A) and protein (B) levels in SVEC4-10 cells and HLECs were detected by quantitative polymerase chain reaction assay and western blot respectively after LYVE-1 CRISPR/Cas9 KO plasmid and siRNA infection. (C) Confocal images of S1PR1 (green) and LYVE-1 (red) in SVEC4-10 and HLEC cells with or without LYVE-1 knockout were stimulated with LMW-HA (10 μg/mL) for 2 h. Nuclei were stained with DAPI (blue). The scale bar represents 10 μm. (D) shows the mean ± SD quantification of the cytosolic intensity of S1PR1 in (C). Data are obtained from at least three independent experiments and represent mean ± SEM. *p < 0.05, **p < 0.01, ***p < 0.001 in Student's t test. Arrows (white) indicate S1PR1.

Based on the above results, we further speculate that LMW-HA may regulate the localization of S1PR1 through LYVE-1. To rigorously test our hypothesis, we initially selectively ablated LYVE-1 expression in both murine and human LECs (Fig. 5A, B), and examined the impact of LYVE-1 on the subcellular localization of S1PR1 upon exposure to LMW-HA stimulation. As expected, our results have demonstrated a significant suppression of LMW-HA-mediated S1PR1 endocytosis in the context of LYVE-1 deficiency (Fig. 5C, D). These results highlight the essential mediatory function of LYVE-1 in the internalization of S1PR1 stimulated by LMW-HA.

### LMW-HA/LYVE-1 pathway induces S1PR1 endocytosis via activating Src phosphorylation

Given the above finding, we proceeded to investigate the intricate mechanisms underlying the regulation of S1PR1 endocytosis by LYVE-1, particularly within the context of LMW-HA. Previous studies have demonstrated that the tyrosine (Tyr) phosphorylation of S1PR1 is a pivotal event initiating the endocytosis process, with Src-mediated phosphorylation identified as a key activator^9^, ^13^. In order to elucidate the potential role of LYVE-1 in modulating the internalization of S1PR1 through a Src activation-dependent mechanism, we first conducted an investigation to assess alterations of phosphorylation levels of Src and S1PR1(Tyr) subsequent to LYVE-1 ablation under LMW-HA stimulation. As expected, upon exposure to LMW-HA, there was a marked elevation in the phosphorylation levels of Src and S1PR1 (Tyr) within wild-type LECs (Fig. 5A-C). However, this increase was notably attenuated following the genetic ablation of LYVE-1(Fig. 5A-C), suggesting that LYVE-1 mediated Src activation and S1PR1 Tyr phosphorylation in response to LMW-HA stimulatiom. Then, to validate that LMW- HA-activated Src contribute to tyrosine phosphorylation of S1PR1, we used an Src kinase inhibitor PP2 to block Src activation. The results showed that the LMW-HA triggered Tyr phosphorylation of S1PR1 could be significantly suppressed by the addition of PP2 (Fig. 5D-F). Furthermore, the inhibitory effect of PP2 extended to the endocytosis of S1PR1 and the lumen formation ability of LECs under LMW-HA stimulation (Fig. 5G-J). Taken together, these results suggest that LMW-HA/LYVE-1 pathway could induce S1PR1 phosphorylation through activation of Src, ultimately leading to S1PR1 endocytosis.

## Discussion

There are five G protein-coupled receptors (S1PR1-5) in binding to their ligand sphingosine-1-phosphate(S1P), of which only S1PR1 has been confirmed to be predominantly expressed in mice and human LECs^2^ (Fig. S2). Although S1PR1 has been implicated in mediating lymphangiogenesis, its exact role and molecular mechanisms remain poorly characterized^3^, ^4^. Extracellular matrix (ECM) components and their dynamic products are active stimulating factors in tumor microenvironment (TME)^6^. We have previously reported that hyaluronan fragments (LMW-HA) abnormally enriched in TME could promote lymphangiogenesis^6^. However, its role in lymphatic endothelial cell regulation remains unknown. In this study, we showed that LMW-HA derived from tumor cells could induce S1PR1 translocation from the LECs surface to the endoplasmic reticulum (ER), leading to lymphangiogenesis promotion. More importantly, we reported a novel mechanism of S1PR1 internalization initiated by LMW-HA binding to lymphatic endothelial hyaluronan receptor 1 (LYVE-1), which in turn activates Src kinase and subsequently increases S1PR1 tyrosine phosphorylation.

Prior research has demonstrated that maintaining vascular homeostasis requires appropriate S1PR1 location and signaling and inflammatory factors like TNF-α induced the endocytosis of S1PR1 form the cell membrane to the ER could destroy the endothelial cell homeostasis^9^. So we first investigated whether the redistribution of S1PR1 occurred in LECs in response to another inflammatory factor LMW-HA and its effect on lymphangiogenesis. In line with our hypothesis, LMW-HA induced the translocation of membrane-bound S1PR1 to the cytoplasm, similar to that increased S1P induced-S1PR1endocytosis^14^. Notably, this process was associated with LMW-HA-induced lymphangiogenic activity, which was significantly reduced by the presence of an endocytosis inhibitor dynasore. Additionally, aligning with previous studies^4^, our study also found that down-regulation of S1PR1 by RNA interference could enhance lymphaitc endothelial cell migration and tube formation. In fact, previous studies reported that S1PR1 could promote lymphangiogenesis by binding to its ligand S1P, while we and others found that S1PR1 knockout could lead to lymphangiogenesis^3^, ^4^. This contradictory result may be attributed to S1PR1 surface retention, which resulted in inhibiting the excess formation of lymphatic vessels. In light of these observations, our data suggested that the LMW-HA could trigger S1PR1 endocytosis, leading to impairing S1PR1 maintained lymphatic homeostasis.

Subsequently, we investigated the molecular mechanism of LMW-HA on S1PR1 endocytosis. Our previous work has demonstrated that LYVE-1, the primary receptor for pericellular matrix hyaluronan in LECs, mediates lymphatic vessel growth upon LMW-HA binding^12^. Meanwhile, S1PR1 is known to interact with CD44, and LYVE-1 exhibits 41% homology with CD44^8^. S1PR3, a related S1P receptor, has been documented to bind CD44 and LYVE-1 in vascular and lymphatic endothelial cells, respectively^7^, ^8^. Based on these, we speculated that LYVE-1 might be involved in LMW-HA-induced S1PR1 endocytosis. As expected, our results revealed that a physical interaction between S1PR1 and LYVE-1, with LYVE-1 knockdown reducing S1PR1 endocytosis, suggesting a novel mechanism of LYVE-1-mediated new lymphatic vessel formation by modulating S1PR1 redistribution.

Although S1PR1 was shown to occur a LYVE-1-dependent endocytosis pathway (dynamin-mediated sorting to endoplasmic reticulum), the detailed mechanisms remain to be clarified. A growing body of literature has indicated that the phosphorylation of S1PR1 at C-terminal serine/threonine residues or ERY motif tyrosine residues is the first step necessary for endocytosis^9^, ^15^. However, only the phosphorylation of S1PR1 at the tyrosine residue Y^143^ triggers its internalization into the ER and protects it from degradation^9^. Our study showed that LMW-HA had little effect on S1PR1 expression (Fig. S3), but could upregulate S1PR1 tyrosine (Tyr) phosphorylation through LYVE-1 under LMW-HA stimulation. It is well acknowledged that Src kinase is able to induce S1PR1 tyrosine phosphorylation^13^. Also, we and other studies have shown that Src phosphorylation is involved in the downstream of the LYVE-1 signaling pathway^7^, ^16^. Together with these findings, we next investigated whether LMW-HA could induce S1PR1 endocytosis through LYVE-1-mediated Src kinase activation. Our results showed that LMW-HA-induced Src phosphorylation could trigger the endocytosis of S1PR1, which could be reversed by knocking out LYVE-1 or PP2 (an Src inhibitor) treatment. Furthermore, we demonstrated that the addition of PP2 could disrupt LMW-HA-induced lymphangiogenesis. Altogether, our study suggested that LMW-HA-induced S1PR1 internalization was attributed to LYVE-1-mediated Src activation and S1PR1 tyrosine phosphorylation.

In summary, this paper demonstrated that tumor cells-derived LMW-HA could trigger S1PR1 endocytosis, which leads to lymphangiogenesis. Notably, we discovered that the LMW-HA/LYVE-1-Src pathway-activated S1PR1 phosphorylation may be the underlined mechanism for S1PR1 LECs redistribution. Our findings unveiled a new role of extracellular matrix in tumor microenvironment in regulating S1PR1 redistribution. The study may present a potential application to therapeutic intervention for tumor lymphangiogenesis.

## Supplementary Material

Supplementary figures and table.

## Figures and Tables

**Figure 6:**
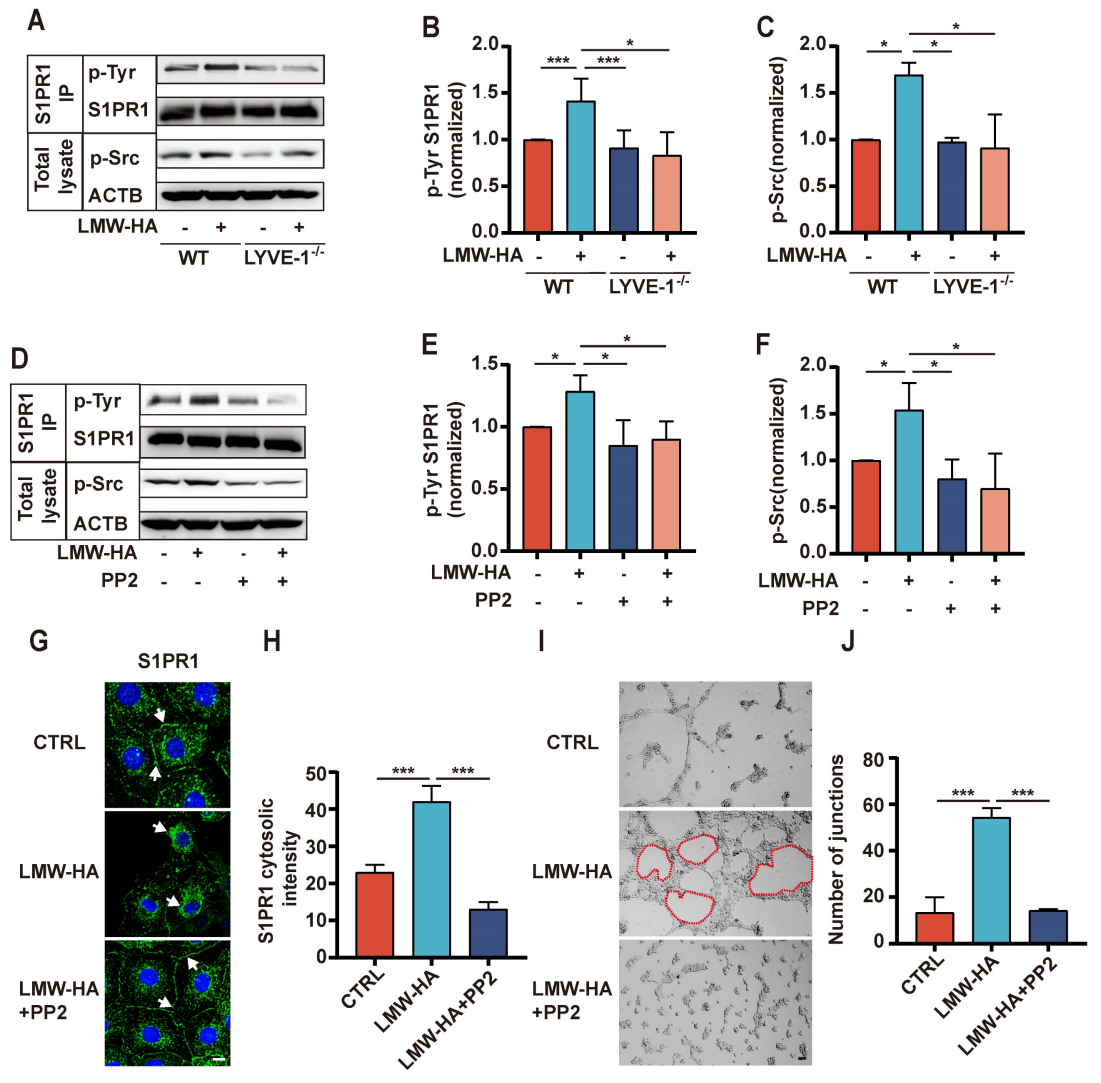
** LMW-HA/LYVE-1 pathway induces S1PR1 endocytosis via activating Src phosphorylation.** (A-C) SVEC4-10 cells with or without LYVE-1 knockout were stimulated with LMW-HA for 30 min. (A, B) Tyrosine phosphorylated S1PR1 was measured by pull-down of S1PR1 using an anti-phospho-Tyr (PY99) antibody. (A, C) Phosphorylated Src kinase was detected by immunoblots using an anti-phospho-Src antibody. β-actin (ACTB) was used as the loading control. (D-F) The cells were treated with PP2 (an Src kinase inhibitor, 10 μM) or vehicle for 30 min before LMW-HA stimulation for 2h. (D) The Tyr phosphorylation level of S1PR1 and Src phosphorylation level were detected as (A). (E, F) The phosphorylated Src kinase and tyrosine phosphorylation bands were quantified by densitometry analysis and normalized against ACTB and S1PR1. (G, H) Representative confocal images (G) and cytosolic intensity quantitative analysis (H) of S1PR1 (green) in SVEC4-10 cells treated with LMW-HA in the presence or absence of PP2. DAPI was stained blue. The scale bar represents 50 μm (I, J) Representative images (I) and quantitative analysis (J) of the tube formation assay of SVEC4-10 cells treated with LMH-HA with or without PP2. The scale bar represents 50 μm. All data from three independent experiments were shown and represent mean ± SEM. *p < 0.05, ***p < 0.001 in one-way ANOVA. Arrows (white) indicate S1PR1.

**Figure 7:**
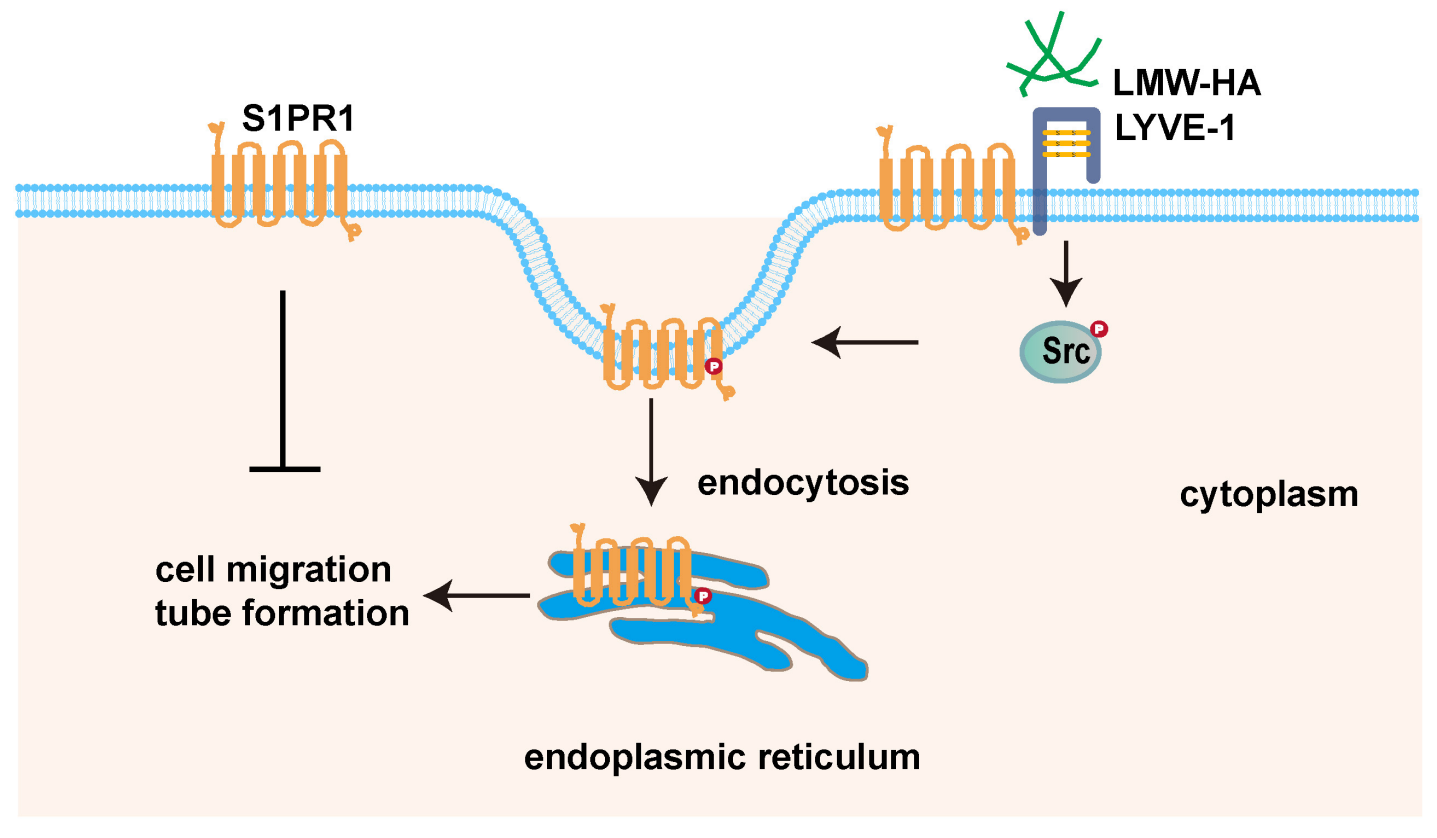
** LMW-HA induces endocytosis of S1PR1 to promote lymphangiogenesis through LYVE-1-Src pathway.** Scheme summarizing the proposed mechanism by which LMW-HA drives cell migration and tube formation. We propose that LMW-HA-induced S1PR1 endocytosis promoted lymphangiogenesis in a LYVE-1/Src/S1PR1 phosphorylation-dependent way.

## Abbreviations

S1PR1: sphingosine-1-phosphate receptor 1
ECM: extracellular matrix
LECs: lymphatic endothelial cells
TME: tumor microenvironment
LMW-HA: low molecular weight hyaluronic acid
LYVE-1: lymphatic endothelial hyaluronan receptor 1
S1P: Sphingosine-1-phosphate
HA: hyaluronic acid
CD44: cluster of differentiation 44
S1PR3: sphingosine-1-phosphate receptor 3
HLECs: human lymphatic endothelial cells
PBS: phosphate buffered saline
FBS: fetal bovine serum
CM: conditioned media
BSA: bovine serum albumin
siRNA: small interfering RNA
ACTB: β-actin
HAS2: hyaluronan synthase 2
INT-HA: intermediate-sized hyaluronan
HMW-HA: high molecular weight hyaluronan
BiP: binding immunoglobulin protein
ER: endoplasmic reticulum
Tyr: tyrosine

## References

[1] Ran S, Volk L, Hall K, Flister MJ (2010). Lymphangiogenesis and lymphatic metastasis in breast cancer. Pathophysiology.

[2] Yoon CM, Hong BS, Moon HG, Lim S, Suh PG, Kim YK (2008). Sphingosine-1-phosphate promotes lymphangiogenesis by stimulating S1P1/Gi/PLC/Ca2+ signaling pathways. Blood.

[3] Zheng Z, Zeng YZ, Ren K, Zhu X, Tan Y, Li Y (2019). S1P promotes inflammation-induced tube formation by HLECs via the S1PR1/NF-kappaB pathway. International immunopharmacology.

[4] Geng X, Yanagida K, Akwii RG, Choi D, Chen L, Ho Y (2020). S1PR1 regulates the quiescence of lymphatic vessels by inhibiting laminar shear stress-dependent VEGF-C signaling. JCI insight.

[5] Bieniasz-Krzywiec P, Martin-Perez R, Ehling M, Garcia-Caballero M, Pinioti S, Pretto S (2019). Podoplanin-Expressing Macrophages Promote Lymphangiogenesis and Lymphoinvasion in Breast Cancer. Cell metabolism.

[6] Du Y, Cao M, Liu Y, He Y, Yang C, Zhang G (2022). Tumor microenvironment remodeling modulates macrophage phenotype in breast cancer lymphangiogenesis. FASEB journal.

[7] Yu M, Zhang H, Liu Y, He Y, Yang C, Du Y (2015). The cooperative role of S1P3 with LYVE-1 in LMW-HA-induced lymphangiogenesis. Experimental cell research.

[8] Singleton PA, Dudek SM, Ma SF, Garcia JG (2006). Transactivation of sphingosine 1-phosphate receptors is essential for vascular barrier regulation. Novel role for hyaluronan and CD44 receptor family. Journal of biological chemistry.

[9] Anwar M, Amin MR, Balaji Ragunathrao VA, Matsche J, Karginov A, Minshall RD (2021). Tyrosine phosphorylation of S1PR1 leads to chaperone BiP-mediated import to the endoplasmic reticulum. Journal of cell biology.

[10] Huang J, Zhang L, Wan D, Zhou L, Zheng S, Lin S (2021). Extracellular matrix and its therapeutic potential for cancer treatment. Signal transduction and targeted therapy.

[11] Gao F, Zhang G, Liu Y, He Y, Sheng Y, Sun X (2022). Activation of CD44 signaling in leader cells induced by tumor-associated macrophages drives collective detachment in luminal breast carcinomas. Cell death & disease.

[12] Wu M, Cao M, He Y, Liu Y, Yang C, Du Y (2015). A novel role of low molecular weight hyaluronan in breast cancer metastasis. FASEB journal.

[13] Chavez A, Schmidt TT, Yazbeck P, Rajput C, Desai B, Sukriti S (2015). S1PR1 Tyr143 phosphorylation downregulates endothelial cell surface S1PR1 expression and responsiveness. Journal of cell science.

[14] Shida D, Inoue S, Yoshida Y, Kodaka A, Tsuji T, Tsuiji M (2016). Sphingosine kinase 1 is upregulated with lysophosphatidic acid receptor 2 in human colorectal cancer. World journal of gastroenterology.

[15] Zhao X, Kiyozuka K, Konishi A, Kawabata-Iwakawa R, Minamishima YA, Obinata H (2023). Actin-binding protein filamin B regulates the cell-surface retention of endothelial sphingosine 1-phosphate receptor 1. Journal of biological chemistry.

[16] Johnson LA, Jackson DG (2021). Hyaluronan and Its Receptors: Key Mediators of Immune Cell Entry and Trafficking in the Lymphatic System. Cells.

