# Supplementary material for: Tumor cell-derived hyaluronan fragments induce endocytosis of S1PR1 to promote lymphangiogenesis through LYVE-1-Src pathway
Source: J Cancer. 2025 Jan 27;16(5):1466–78. doi: 10.7150/jca.104309 (PMC11843240; doi:10.7150/jca.104309)
Supplement: Supplementary file 1 — Supplementary figures and table. [file jcav16p1466s1.pdf]

Supplementary Figure

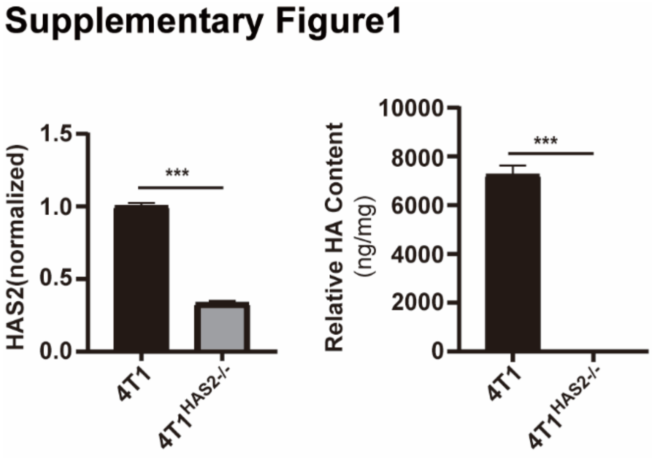

Supplementary Figure 1. The expression level of hyaluronan acid synthase 2 (HAS2). The expression level of hyaluronan acid synthase 2 (HAS2) in 4T1 and 4T1$^{HAS2-/-}$ cells, and the relative amount of HA in the conditioned medium dramatically decreased after HAS2 knocking out.

Supplementary Figure 2

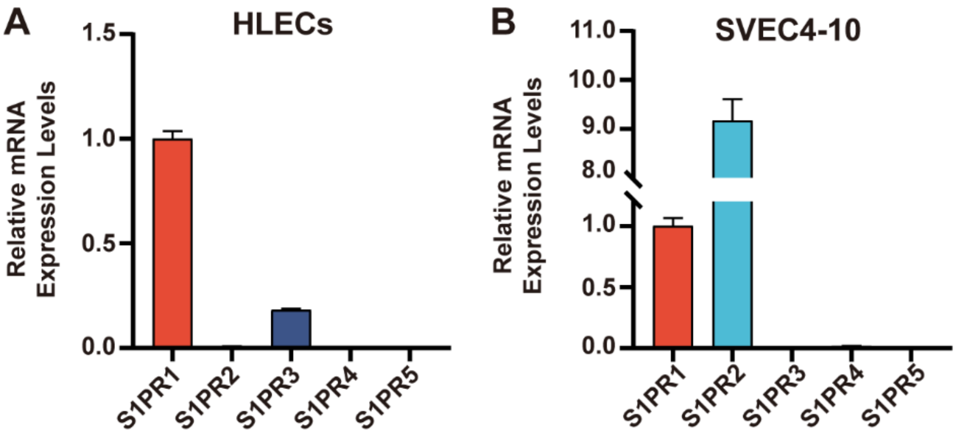

Supplementary Figure 2. Expression patterns of five S1P receptors in LECs. The expression of S1PR1-5 in HLECs (A) and SVEC4-10 (B) was measured by RT-PCR.

Supplementary Figure 3

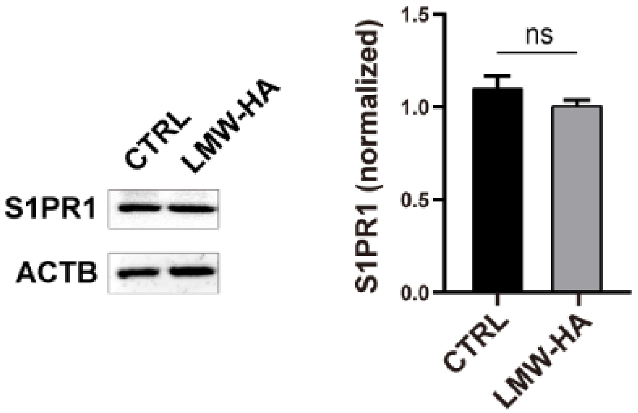

Supplementary Figure 3. LMW-HA had little effect on S1PR1 expression. S1PR1 was detected by immunoblots after treated as indicated. β-actin was used as the loading control.

Supplementary Table 1. The sequence information of primers is as followed.

	primer	sequence（5'—3'）
human	S1PR1-F	CCACAACGGGAGCAATAACT
	S1PR1-R	CAGAATGACGATGGAGAGCA
	S1PR2-F	CCAAGCATTATGTGCTGTGC
	S1PR2-R	CAGAAGGAGGATGCTGAAGG
	S1PR3-F	TGATTGTGGTGAGCGTGTTCA
	S1PR3-R	GGCCACATCAATGAGGAAGAG
	S1PR4-F	CCAAGCGCTACATCCTCTTC
	S1PR4-R	CAGAGGTTGGAGCCAAAGAC
	S1PR5-F	GGTCATCGTCCTGCATTACA
	S1PR5-R	AGCAGATCCGACAACGTGA
	β-actin-F	TCTACAATGAGCTGCGTGTG
	β-actin-R	ATGGCTGGGGTGTTGAAG
mouse	S1PR1-F	TCTGCTCCTGCTTTCCATCG
	S1PR1-R	AGGATGTCACAGGTCTTCGC
	S1PR2-F	TTCTGGAGGGTAACACAGTGGT
	S1PR2-R	ACACCCTTTGTATCAAGTGGCA
	S1PR3-F	ACCGCGTGTTCCTTCTGATT
	S1PR3-R	TTGACCAGGCAGTAGATGCG
	S1PR4-F	GTCAGGGACTCGTACCTTCCA
	S1PR4-R	GATGCAGCCATACACACGG
	S1PR5-F	GCTTTGGTTTGCGCGTGAG
	S1PR5-R	GGCGTCCTAAGCAGTTCCAG
	β-actin-F	CCCTCTGAACCCTAAGGCCA
	β-actin-R	GGGACAACACAGCCTGGATG
